# SOCS1 Regulates the Immune Modulatory Properties of Mesenchymal Stem Cells by Inhibiting Nitric Oxide Production

**DOI:** 10.1371/journal.pone.0097256

**Published:** 2014-05-14

**Authors:** Lei Zhang, Rui-Jie Dang, Hong Li, Ping Li, Yan-Mei Yang, Xi-Min Guo, Xiao-Yan Wang, Nan-Zhu Fang, Ning Mao, Ning Wen, Xiao-Xia Jiang

**Affiliations:** 1 Institute of Basic Medical Sciences, Beijing, China; 2 Yanbian University, Yanji City, Jilin Province, China; 3 Chinese PLA General Hospital, Beijing, China; Josep Carreras Leukaemia Research Institute, University of Barcelona, Spain

## Abstract

Mesenchymal stem cells (MSCs) have been shown to be highly immunosuppressive and have been employed to treat various immune disorders. However, the mechanisms underlying the immunosuppressive capacity of MSCs are not fully understood. We found the suppressor of cytokine signaling 1 (SOCS1) was induced in MSCs treated with inflammatory cytokines. Knockdown of SOCS1 did not bring much difference on the proliferation and differentiation properties of MSCs. However, MSCs with SOCS1 knockdown exhibited enhanced immunosuppressive capacity, showing as inhibiting T cell proliferation at extremely low ratio (MSC to T) in vitro, significantly promoting tumor growth and inhibiting delayed-type hypersensitivity response in vivo. We further demonstrated that SOCS1 inhibited the immunosuppressive capacity of MSCs by reducing inducible nitric oxide synthase (iNOS) expression. Additionally, we found the significantly lower SOCS1 expression and higher nitric oxide (NO) production in MSCs isolated from synovial fluid of rheumatoid arthritis patients. Collectively, our data revealed a novel role of SOCS1 in regulating the immune modulatory activities of MSCs.

## Introduction

Mesenchymal stem cells (MSCs) exist in almost all tissues and have been isolated from bone marrow, adipose tissue, umbilical cord, placenta and amniotic fluid [1]–[3]. MSCs possess specific cell surface markers and can differentiate into osteoblast, adipocyte and chondrocyte [4]. Since their initial isolation, MSCs have been investigated for their multipotent differentiation potential for regenerative medicine [5].

Recent studies have found that MSCs have potent immunoregulatory capacity and possess therapeutic potential for various inflammation related diseases [6], [7]. These cells can block T cell proliferation and B cell terminal differentiation, NK cell cytotoxicity, and dendritic cell maturation [8]–[11]. The mechanisms of MSC-mediated immunosuppression are under intensive investigations, and various molecules including indoleamine 2,3-dioxygenase (IDO), inducible nitric oxide synthase (iNOS), prostaglandin E2 (PGE2), transforming growth factor-β (TGF-β), and programmed death-1 ligand (PD-L1) have been shown to be responsible [12]. Moreover, further studies revealed that the immunosuppressive property of MSCs is not innate but rather induced by inflammatory cytokines produced by inflammatory cells [13]. In spite of previous intensive studies on the MSC immunosuppressive function, the detailed mechanisms are still not fully understood.

The suppressor of cytokine signaling 1 (SOCS1) controls signaling of many cytokines, including IFNγ, IFNα, IL-10, IL-12, IL-15, and IL-21 with a feedback loop mechanism [14]. The expression of SOCS1 is upregulated when a cytokine binds to its receptor. SOCS1 then inhibits Janus tyrosine kinase (JAK) activity by binding to the catalytic site of JAK and recruiting the ubiquitin-transferase complex to target JAK for proteasomal degradation [15]. SOCS1 also controls signal transducers and activators of transcription (STAT) and indirectly, toll like receptor (TLR) signaling [16], [17]. Dendritic cells (DCs) are professional antigen-presenting cells with key regulatory roles in the maintenance of tolerance to self-antigens and in the activation of innate and adaptive immunity [18]. Previous studies have shown that SOCS1 is induced in DCs and silencing of SOCS1 enhances antigen presentation by DCs and antigen-specific anti-tumor immunity [19]. Downregulating the production of SOCS1 in DCs allows increased signaling to T cells and resulting in expanded memory T cell populations. Macrophages comprise a heterogeneous population of cells that play an important role in tissue homeostasis as well as coordinating almost all aspects of inflammation [20], [21]. It has been shown that SOCS1 is crucial for IL-4-induced M2 macrophage characteristics, including a high arginase I: iNOS activity ratio, suppression of T cell proliferation, attenuate responses to IFNγ and lipopolysaccharides (LPS) [22]. Together, SOCS1 is not only as a feedback inhibitor of inflammation but also as a critical molecular switch that tunes key signaling pathways to effectively program different sides of the macrophage balance. Since MSCs are responsive to proinflammatory cytokines and regulate immune cells, we hypothesize that SOCS1 may play a role in the immunoregulatory function of MSCs.

In this study, we found that SOCS1 was induced in MSCs by inflammatory cytokines. SOCS1 inhibited iNOS expression in cytokine-activated MSCs. Downregulating the production of SOCS1 in MSCs allowed increased nitric oxide (NO) production and then enhanced immunosuppressive capacity of MSCs. Therefore, our study revealed a novel role for SOCS1 in regulating the immunoregulatory property of MSCs.

## Materials and Methods

### Ethics Statement

All animal experimental protocols of the study are in accordance with the national guidelines for the use of animals in scientific research. It's also approved by Animal Care and Use Committee of Beijing Institute of Basic Medical Sciences, with the approval number BMS-1104139.

### Patients Characteristics

The synovial fluid of rheumatoid arthritis (RA) patients and osteoarthritis (OA) patient was obtained at Chinese PLA General Hospital (Beijing, China). The institutional ethics committee of Chinese PLA General Hospital approved the study, and all patients gave written informed consent.

### Mice

C57BL/6 mice were purchased from the Laboratory Animal Center, Academy of Military Medicine Sciences, Beijing, China, and maintained under specific pathogen-free conditions. Animals were matched for age and gender.

### Cells

Primary murine MSCs (MSCs) derived from murine bone and bone marrow were isolated and cultured as described in our previous report [23]. The murine MSC line C3H10T1/2 (CCL-226™) cells were obtained from ATCC and grown in minimal essential medium (MEM, Gibco) with 4 mM L-glutamine, 100 U/ml penicillin, and 100 U/ml streptomycin and 10% fetal bovine serum (FBS) in a humidified atmosphere of 5% CO_2_ at 37°C.

Human bone marrow-derived MSCs (BMMSC) were isolated from healthy donor as described before [11]. Synovial fluid (SF) samples were obtained from knee effusion of 6 rheumatoid arthritis (RA) patients and 1 osteoarthritis (OA) patient. Synovial Fluid Mononuclear Cells (SFMC) were isolated by density gradient centrifugation on Ficoll-paque™-Plus (GE Healthcare, Amersham, UK) and cultured in human MSC medium (ExCell Biology, Inc.).

### Flow cytometry

Antibodies against mouse CD45, CD105, CD44, CD29, CD11b, and Sca-1 were from BioLegend. BrdU flow kit was from BD Pharmingen (SanDiego, CA, USA). Data were collected on a FACS Canto II (BD) and were analyzed with FlowJo software (TreeStar).

### Transduction

C3H10T1/2 were seeded the day before transduction in serum and antibiotic-free medium. The next day, C3H10T1/2 were transduced with lentivirus expressing murine SOCS1 shRNA (MSC/SOCS1sh) or control lentivirus (MSC/CTLsh) (Shanghai GeneChemCo.,Ltd, China) in the presence of 10 ug/ml polybrene (Santa Cruz). Stable transduction clones were obtained by FACS selection.

### Cell proliferation assay

Cell proliferation was measured by cell counting analysis, Cell Counting Kit-8 (CCK-8, Fluka) assay and BrdU incorporation. For cell counting, cells at 4000/well were seeded in 24-well plate in cultured medium. Cells were counted every day for 7 days. The mean cell number was calculated for each triplicate. For CCK-8 assay, cells were suspended at a final concentration of 5000/well in 100 µl medium and cultured in 96-well flatbottomed microplate. CCK-8 (10 µl/well) was added to each well containing 100 µl of culture medium, and then did following the reagent protocol. For BrdU incorporation, cells (1×10^5^/well) were seeded in 6-well plate, 10 µM BrdU (BD) was added and incubated for 2 hours, and then did according to the product protocol.

### In vitro differentiation

For in vitro differentiation, cells were induced with osteogenic induction media containing 0.1 µM dexamethasone, 50 µM ascorbate-2 phosphate,10 mM glycerophosphate (Sigma). To induce adipogenic differentiation, cells were cultured in adipogenic induction media containing 1 µM dexamethasone, 200 µM indomethacin, 0.5 µM 3-isobutyl-1-methyl-xanthine and 10 ug/mL insulin (Sigma). Alkaline phosphatase (ALP) assay and Oil-Red-O staining were performed as described previously [23].

### CFSE staining

CD3^+^T cells selected with CD3ε MicroBead Kits (Miltenyi Biotec) were labeled with 5 µM carboxy fluorescein diacetate succinimidyl ester (CFSE, Invitrogen) for 7 min at room temperature. Labeling was terminated according to the manufacture's protocol. After washing, cells were activated with 50 ng/ml PMA,1 µg/ml ionomycin (Sigma) for 24 h, and then cultured with MSC/SOCS1sh or MSC/CTLsh. Cell division, as evidenced by reduction of fluorescence intensity, was analyzed by flow cytometry.

### Detection of NO

MSCs were stimulated with IFNγ and TNFα (R&D). NO in culture supernatants was detected using a modified Griess reagent (Sigma-Aldrich). Briefly, all NO_3_ was converted into NO_2_ by nitrate reductase, and total NO_2_ detected by the Griess reaction.

### Real-time PCR

Total RNA was extracted with TRIZOL (Sigma) and reverse-transcribed into cDNA with reverse transcriptase kit (Takara). cDNA was used as template in real-time PCR with SYBR Green reagent from TOYOBO (Shanghai, China) to determine specific gene expression. Primer sequences were as follows: mouse β-actin, CTTCCGCCTTAATACTTC (forward) and AAGCCTTCATACATCAAG (reverse); mouse SOCS1, CAACGGAACTGCTTCTTC (forward) and AAGGCAGTCGAAGGTCTC (reverse); mouse iNOS CAGCTGGGCTGTACAAACCTT (forward) and CATTGGAAGTGAAGCGTTTCG (reverse); mouse RUNX2, CCACAAGGACAGAGTCAGAT (forward) and GATAGGAGGGGTAAGACTGG (reverse); mouse Osteocalcin (OC), GGGCAATAAGGTAGTGAACA (forward) and GTCTTCAAGCCATACTGGTC (reverse); human SOCS1 GATGGTAGCACACAACCAG (forward) and AGGAAGAGGAGGAAGGTTCT (reverse).

### Western blot

Protein samples in SDS sample buffer were heated and separated on SDS-polyacrylaminde gel. Then proteins were electroblotted to PVDF transfer membranes and revealed by antibodies against SOCS1, iNOS or β-ACTIN (Cell Signaling Technology, Inc.) overnight at 4°C. Finally, the blot was subjected to chemiluminescent detection according to the manufacturer's instructions.

### Mouse melanoma model

B16-F0 mouse melanoma cells were expanded in complete α-MEM medium in vitro. Each mouse was injected with 5×10^5^ B16-F0 cells in 100 µl PBS subcutaneously on the left back, with or without co-injection of MSC/SOCS1sh or MSC/CTLsh (1×10^6^ cells). The MSC cells were again administrated at day 3, 6 and 9 at the same sites, with PBS serving as a control. Mice were observed daily and euthanized 16 days later, when tumor began to significantly affect mobility. The melanoma tumors were then excised and weighed.

### DTH response

C57BL/6 mice (8-10 weeks old) were immunized by tail-base injection of OVA (10 µg in 50 µl saline) emulsified with 50 µl complete Freund's adjuvant. DTH was tested after 6 days, by challenging with 200 µg aggregated OVA in 30 µl saline injected into the right hind footpad. The left footpad was injected with 30 µl of saline with MSCs or without MSCs as a negative control. After 24 h, antigen-induced footpad thickness increment was measured using a caliper (no. 7308, Mitutoyo, Tokyo, Japan) and calculated as: thickness increment  = R-L, where R and L are thickness of right and left footpads, respectively.

### Statistical analysis

Data are presented as mean ±S.D. Statistical significance was assessed by unpaired two-tailed Student's t-test.

## Results

### Inflammatory cytokines mediated induction of SOCS1 in MSCs

**Figure 1:**
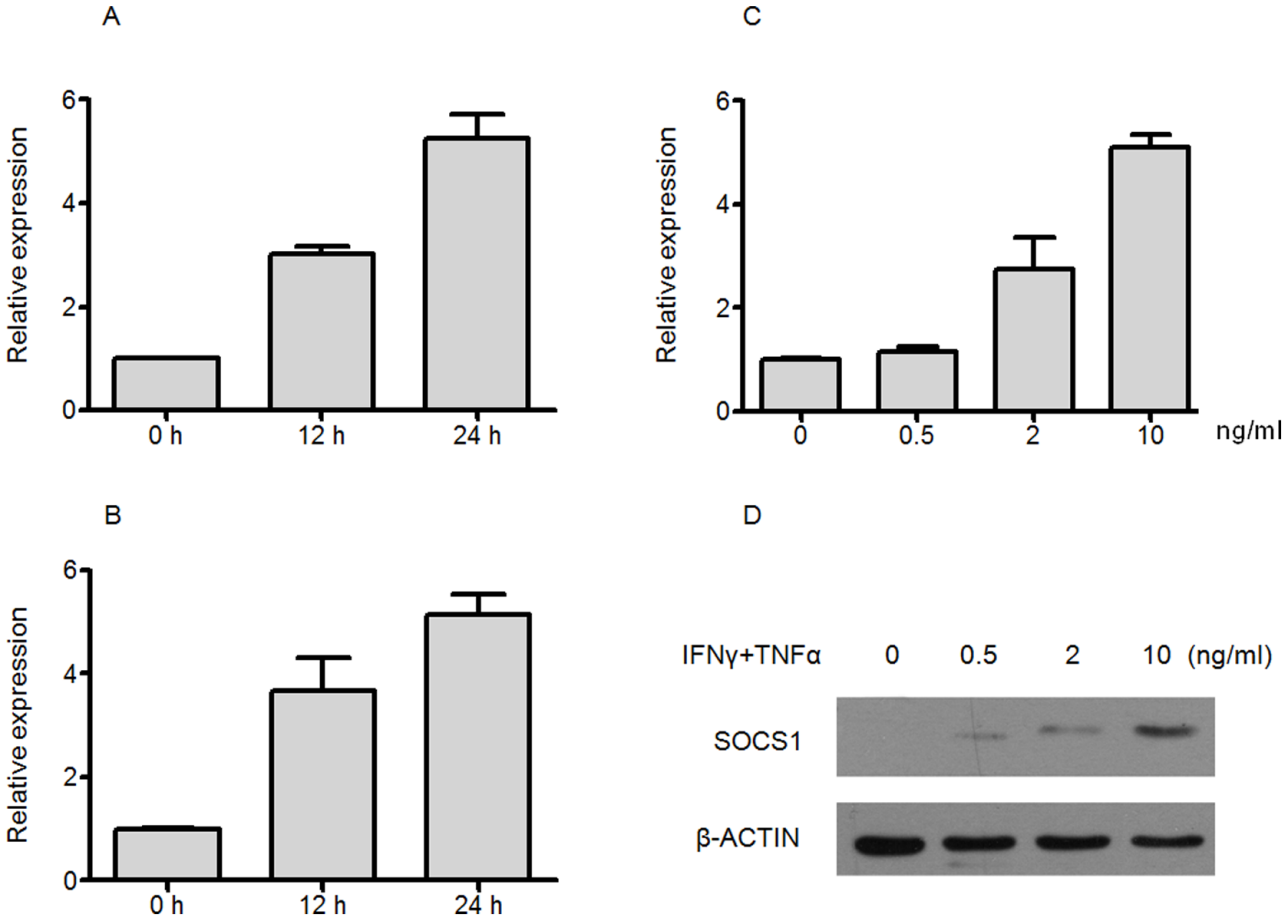
Inflammatory cytokines induce SOCS1 expression in MSCs. MSCs (A) and C3H10T1/2 (B) were treated with IFNγ plus TNFα (2 ng/ml each) for 12 h and 24 h, and cells were collected in Trizol. SOCS1 mRNA expression was determined with real-time PCR. C3H10T1/2 were treated with indicated concentration of IFNγ plus TNFα for 24 h, SOCS1 mRNA level (C) was determined by real-time PCR and protein level (D) was determined by western blot.

SOCS1 has been described as being up-regulated in various antigen presenting cells, like DCs and macrophage, and contributing to the modulation of the immune response mediated by these cells. We investigated the expression of SOCS1 in primary cultured mouse MSCs and murine MSC cell line, C3H10T1/2, which has been employed in a lot of in vitro and in vivo studies. To establish a time-course for this event, changes in SOCS1 levels were monitored by real-time PCR at different time-points (12 h and 24 h), following stimulation of cells with inflammatory cytokines IFNγ plus TNFα at 2 ng/ml each. As shown in Figure 1A and Figure 1B, SOCS1 was significantly induced in MSCs and C3H10T1/2 compared to their non-treated cells. Treatment of C3H10T1/2 with increasing concentration of inflammatory cytokines IFNγ plus TNFα (0.5, 2 and 10 ng/ml each) showed a significant dose-dependent induction of SOCS1 expression, which reached a 5-fold increase in SOCS1 mRNA levels for the highest inflammatory cytokine tested (Figure 1C). To confirm the results obtained by real-time PCR, western blot assay was performed. The protein level of SOCS1 also showed significant increase with the stimulation (Figure 1D).

All these data showed that under inflammatory conditions, SOCS1 expression increases not only in primary cultured mouse MSCs but also in MSC cell line C3H10T1/2. Since the responses of mouse MSCs and MSC cell line, C3H10T1/2 to inflammatory factors are similar and cell line is easier to obtained with high yield for in vitro and in vivo studies, so the subsequent studies were performed in MSC cell line, C3H10T1/2 and referred as MSCs.

### Properties of MSCs with SOCS1 knockdown

**Figure 2:**
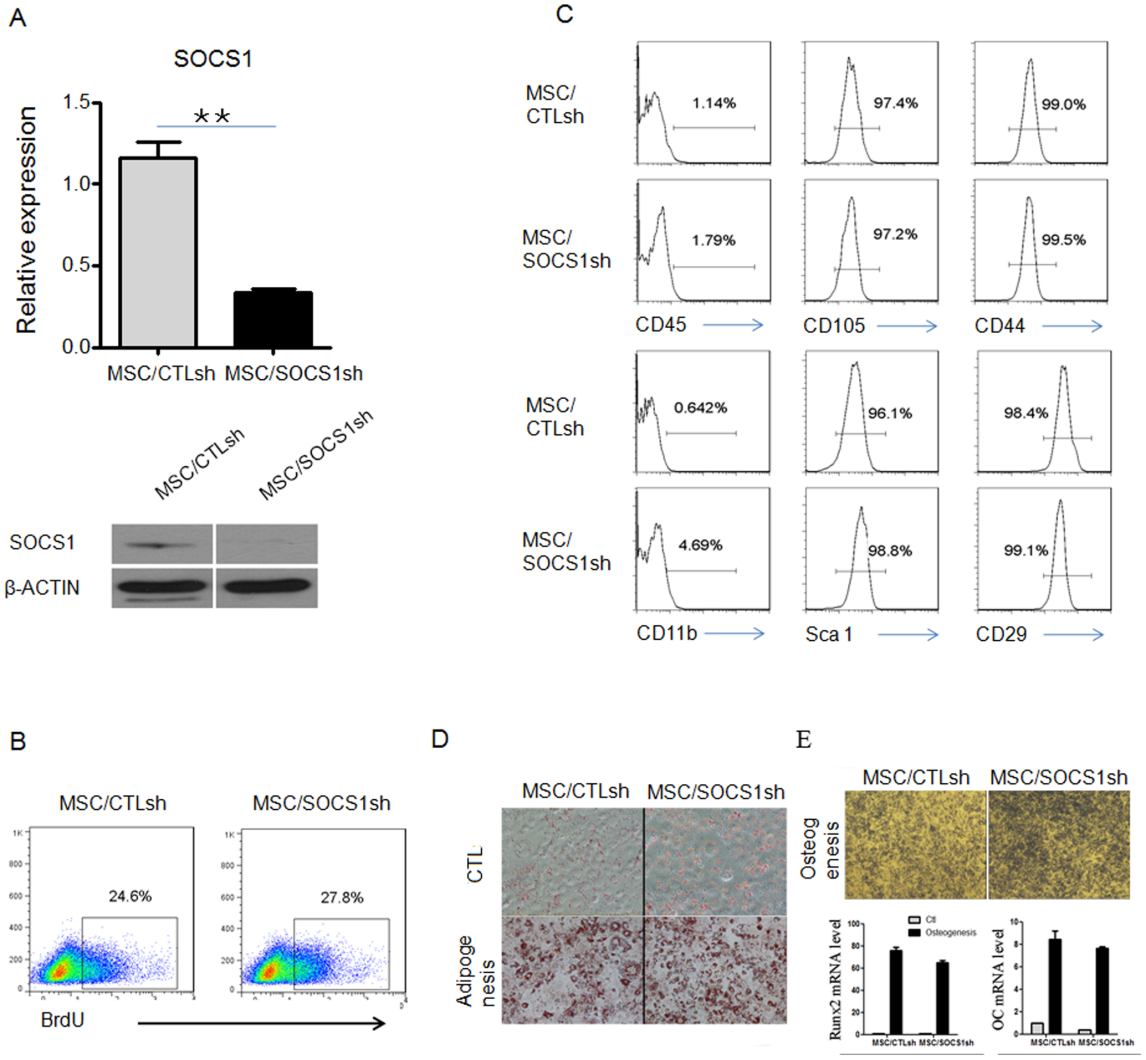
Properties of MSCs after SOCS1 knockdown. A. The knockdown of SOCS1. SOCS1 mRNA (top) was determined by real-time PCR, and protein expression (bottom) was examined by western blot. ^**^P<0.01. B. BrdU incorporation was assessed by flow cytometry analysis. Numbers adjacent indicate percentage of cells in the gate. C. Cell surface marker of MSC/CTLsh, MSC/SOCS1sh was examined by flow cytometry analysis. Numbers above the bar indicate percents of cells in the gate. D. Adipocyte differentiation was examined by Oil-red-O staining. E. Osteoblast differentiation was detected by alkaline phosphatase (ALP) staining (top), Runx2 and Osteocalcin (OC) expression after osteogenic induction were tested by real-time PCR data (bottom).

Since SOCS1 was induced by inflammatory factors, we investigated whether SOCS1 contributed to the immune modulation function of MSCs. Lentivirus expressing SOCS1 shRNA were generated to knock down SOCS1. MSCs transduced with lentivirus were analyzed for expression of green fluorescent protein (GFP). Green fluorescence was found on almost all the cells after transduction and selection by flow cytometry (data not shown). Real-time PCR and western blot analysis showed that SOCS1 was significantly reduced in MSCs expressing SOCS1 shRNA (MSC/SOCS1sh) compared with MSCs expressing scramble shRNA (MSC/CTLsh) (Figure 2A). All these demonstrated the efficiency of our knockdown protocol.

We next analyzed the effect of SOCS1 silence in MSCs. To examine the effect of SOCS1 on MSC proliferation, cell counting, CCK-8 assay and BrdU incorporation were performed. When cultured over several passages, MSC/SOCS1sh did not show a significant increase in their proliferative activity compared with MSC/CTLsh (Figure S1). Moreover, BrdU incorporation data confirmed that SOCS1 silence did not cause significant alterations to their proliferation ability (Figure 2B). MSCs have been shown to be positive for CD105, CD44, CD29, Sca-1 and negative for CD45, CD11b. Our flow cytometry data showed that MSC/SOCS1sh and MSC/CTLsh comprised the similar immunophenotype (Figure 2C).

MSCs can be differentiated into adipocytes and osteoblasts in vitro depending on the culture conditions. When incubated with adipogenic induction media containing dexamethasone, indomethacin, 3-isobutyl-1-methyl-xanthine, insulin and allowed to differentiate, both MSC/SOCS1sh and MSC/CTLsh showed normal lipid accumulation as measured by Oil red O staining (Figure 2D). When stimulated by culture medium containing dexamethasone, glycerophosphate, and ascorbate-2 phosphate, MSCs can be induced to undergo osteogenic differentiation in vitro. After osteogenic induction, both MSC/SOCS1sh and MSC/CTLsh showed enhanced alkaline phosphatase (ALP) activity, an early marker of osteoblast differentiation (Figure 2E top). Analysis of expression of the differentiation markers Runx2 and Osteocalcin confirmed the similarity in the osteogenic processes (Figure 2E bottom).

### SOCS1 knockdown MSCs showed enhanced immunosuppressive capacity in vitro and in vivo

**Figure 3:**
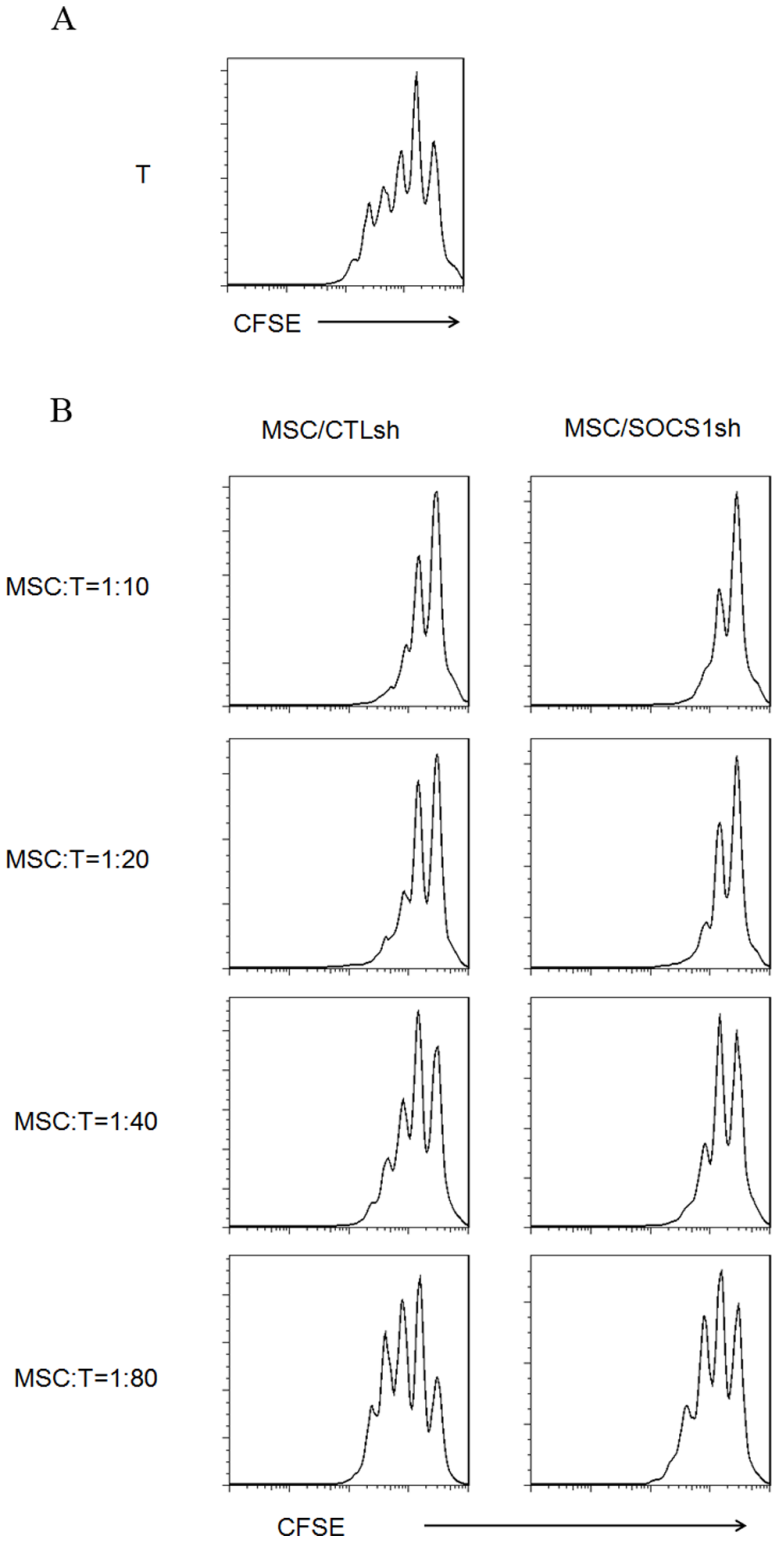
Enhanced inhibition of T cell proliferation by MSC/SOCS1sh. CD3^+^ T cells were isolated from murine spleen with CD3ε MicroBead Kits and labeled with CFSE. Then T cells were stimulated with PMA (50 ng/ml) plus ionomycin (1 ug/ml) for 24h, and then cultured alone (A) or with MSC/CTLsh or MSC/SOCS1sh at different ratios (MSCs to T cells) (B). After 48 h, all the cells were subjected to flow cytometry for T-cell proliferation detection as indicated by the reduction in CFSE intensity. Data are representative of three independent experiments.

We next examined the immunoregulatory function of MSCs with SOCS1 knockdown. Since SOCS1 functions as a negative regulator of signaling by various cytokines, such as IFNγ, IL-12 by inhibiting the Janus kinases (JAKs) in immune cells, we hypothesize the immune-enhancing activity of MSC/SOCS1sh. To test this hypothesis, T cells were stained with CFSE and stimulated with PMA and inomyosin, and then MSC/SOCS1sh and MSC/CTLsh were added at various ratios. As expected, T cell proliferation was inhibited as indicated by the slower reduction in CFSE intensity by MSC/CTLsh. However, instead of stimulating T cell proliferation, MSC/SOCS1sh exhibited stronger immunosuppressive activity. T cell proliferation was strikingly inhibited by MSC/SOCS1sh at ratios as low as 1∶80 (MSCs to T cells) (Figure 3).

**Figure 4:**
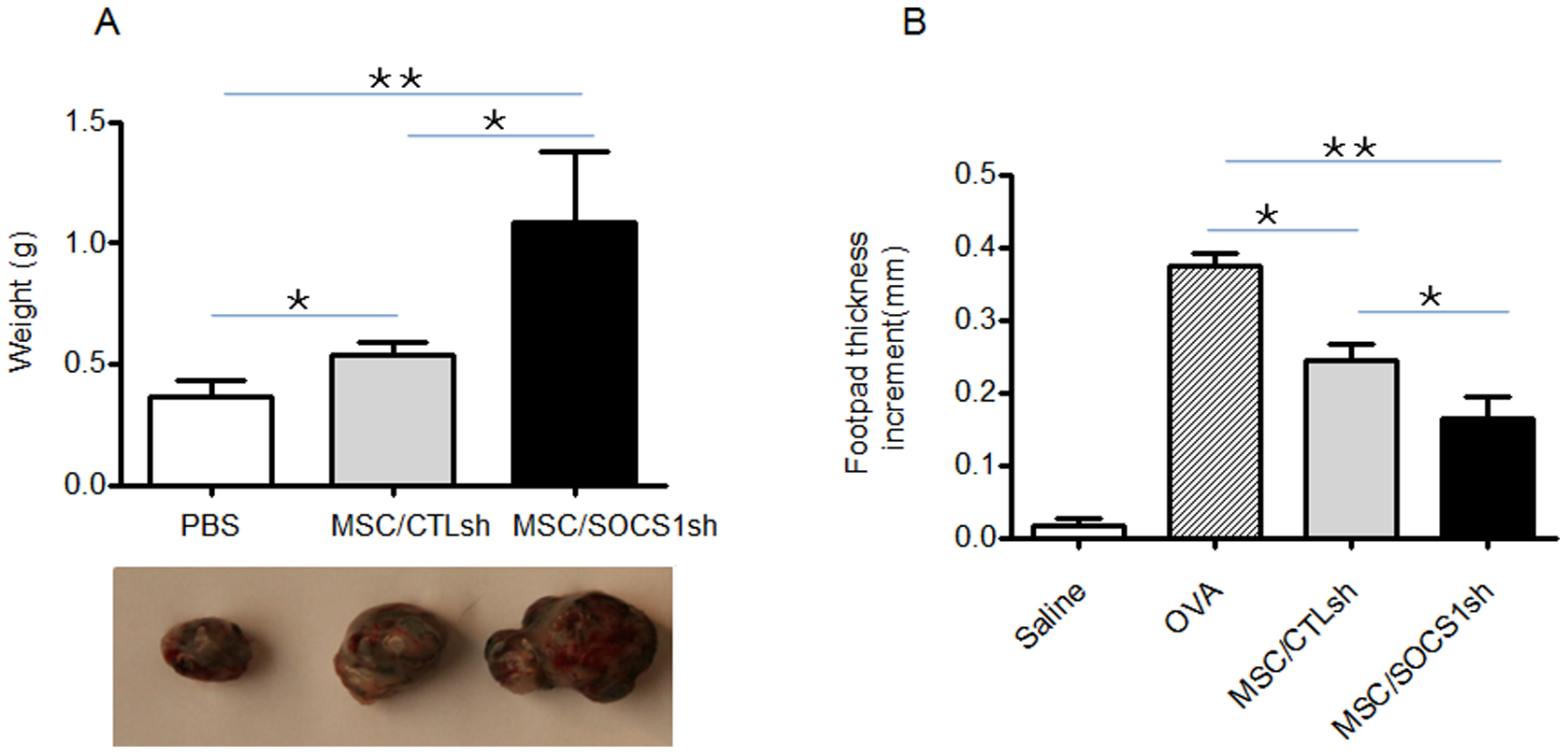
Knockdown of SOCS1 enhance immunosuppressive capacity of MSCs *in vivo*. A.SOCS1-deficient MSCs boost melanoma growth. At day1, B16-F0 cells (5×10^5^ cells) were administrated subcutaneously to the back of C57BL/6 mice, with or without co-injection of MSC/CTLsh and MSC/SOCS1sh, each at the dose of 1×10^6^ cells per mouse. Both types of MSCs were further administrated at day 3, 6 and 9 at the same sites with the original injection, with PBS as a control. 16 days later, the mice were killed and the tumors were weighed (top), picture of the representative tumor of each group was taken under microscope (bottom). Data shown are mean ±S.D. of a representative of 3 independent experiments. ^*^P<0.05 and ^**^P<0.01. B. SOCS1-deficient MSCs inhibit delayed-type hypersensitivity (DTH). C57BL/6 mice were immunized with OVA (10 ug) emulsified with 50 ul complete Freund's adjuvant by tail base injection. On day 7, mice were challenged in the footpad with 200 ug OVA administered without or with MSC/CTLsh or MSC/SOCS1sh (2.5×10^5^ cells). Footpad thickness increment was examined after 24 h as a measure of DTH. Data shown are mean±S.D. of a representative of 3 independent experiments. ^*^P<0.05 and ^**^P<0.01.

The immune response is critical in controlling tumor growth. As MSC/SOCS1sh showed stronger immunosuppressive activity in vitro, we tested their effect in vivo in a mouse model of melanoma. B16-F0 melanoma cells were co-administered with MSC/SOCS1sh or MSC/CTLsh and 16 days later, the resultant tumors were weighed. Both MSCs were found to promote tumor growth, and MSC/SOCS1sh exhibited stronger effect (Figure 4A). One of the striking effects of immunosuppression by MSCs is the ability to suppress DTH response. Then we further tested the immunosuppressive effect in the DTH response. OVA-immunized mice were injected in the footpad with OVA alone or OVA with MSC/SOCS1sh or MSC/CTLsh, and the resultant DTH response measured by footpad thickness increment. As expected, administration of MSC/CTLsh resulted in reduced inflammation. Similar to the effect in melanoma model, MSC/SOCS1sh exhibited stronger immunosuppressive effect, showing as sharp reduction of footpad thickness increment (Figure 4B). Collectively, MSCs with SOCS1 knockdown showed enhanced immunosuppressive activity in vitro and in vivo.

### SOCS1 negatively regulates iNOS expression in inflammatory cytokine-stimulated MSCs

**Figure 5:**
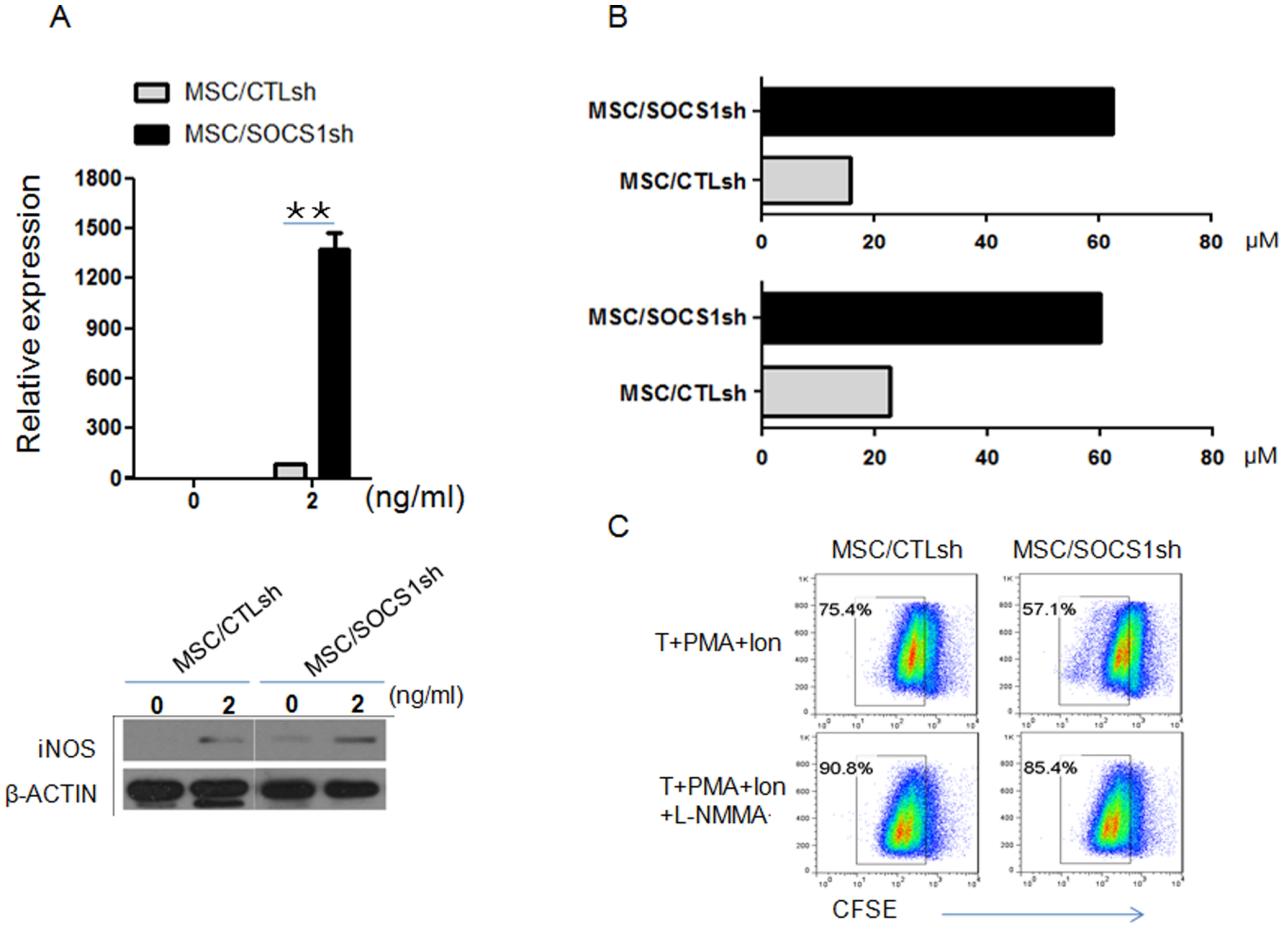
SOCS1 negatively regulates iNOS expression and NO production in MSCs. A. iNOS expression was examined by real-time PCR (top) and western blot (bottom). MSC/CTLsh or MSC/SOCS1sh were treated with or without IFNγ plus TNFα (2 ng/ml each) for 24 h. ^**^P<0.01. B. MSC/CTLsh or MSC/SOCS1sh were treated without (top) or with (bottom) irradiation, then stimulated with IFNγ plus TNFα (2 ng/ml each) for 24 h. The supernatant was examined for NO production by Griess assay. C. CD3^+^ T cells were stimulated with PMA (50 ng/ml) plus ionomycin (1 ug/ml) for 24 h, and then cultured with MSC/CTLsh or MSC/SOCS1sh at 1∶20 ratio (MSC: T), L-NMMA (1 mM) was added at the beginning of co-culture. After 48 h, all the cells were subjected to flow cytometry for T-cell proliferation detection as indicated by the reduction in CFSE intensity. Numbers adjacent indicate percentage of cells in the gate.

Previous studies [7], [8] have shown that proinflammatory cytokines can stimulate mouse MSCs to express iNOS and produce NO, which can inhibit the proliferation of T lymphocytes. And their ability to produce NO in the presence of proinflammatory did not change with passaging. To assay the efficacy of inflammatory cytokines to induce NO secretion by MSCs, we added graded concentrations of IFNγ plus TNFα (0.5, 2 and 10 ng/ml each) to MSCs. We found that MSCs were highly responsive to low levels of cytokines; significant induction of NO was observed when as little as 0.5 ng/ml of each cytokine was added (data not shown). Real-time PCR showed the increased iNOS accumulation with the stimulation of graded concentrations of IFNγ plus TNFα (data not shown). Next we examined the iNOS expression after SOCS1 knockdown. iNOS mRNA and iNOS protein expression were examined by real-time PCR and western blotting analysis respectively. Figure 5A showed the significant increase of iNOS mRNA and iNOS protein expression in MSC/SOCS1sh. The supernatant from MSC/SOCS1sh and MSC/CTLsh stimulated with inflammatory cytokines was examined for their nitrate concentration. As shown in Figure 5B, NO production was increased dramatically after SOCS1 knockdown, no matter MSCs irradiated or not. To further confirm the NO-dependent manner of the strong immunosuppressive function of MSC/SOCS1sh, the iNOS inhibitor NG-monomethyl-L- arginine acetate salt (L-NMMA) was added to the T cell proliferation assay. As expected, L-NMMA restored T-cell proliferation in the co-culture assay (Figure 5C). Therefore, SOCS1 might attenuate the immunosuppressive capacity of MSCs by inhibiting iNOS expression and thereby NO release.

### Low SOCS1 expression in MSCs from synovial fluid of RA patient

**Figure 6:**
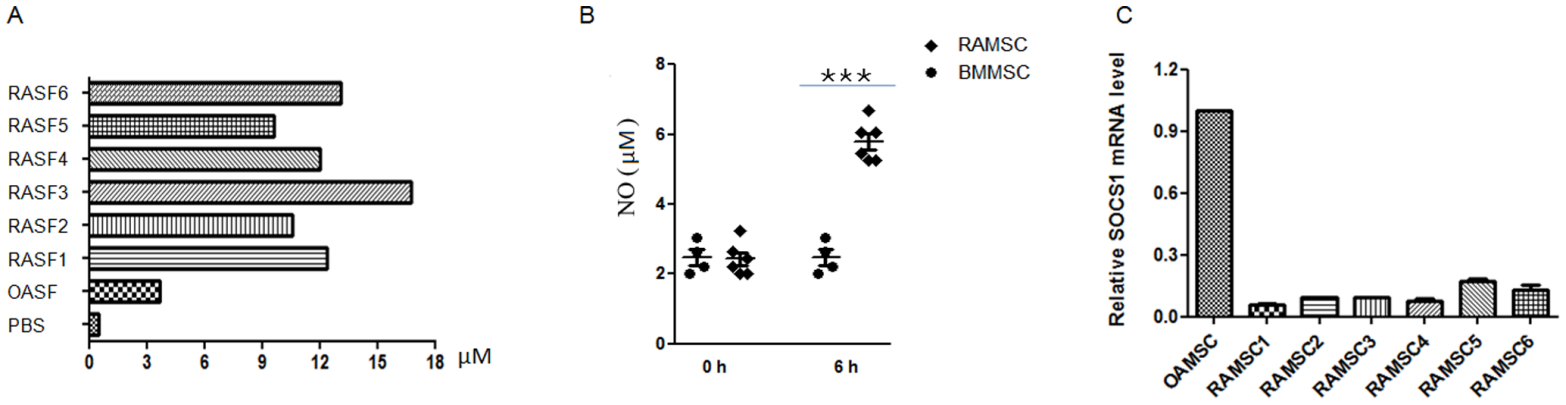
NO secretion and SOCS1 expression in RAMSC. A. NO secretion in RA synovial fluid (RASF) or OA synovial fluid (OASF) was examined by Griess assay. B. RAMSC or BMMSC were seeded in 24-well plate (5×10^4^/well) and stimulated with IFNγ plus TNFα (2 ng/ml each) for 6 h. The supernatant was examined for NO production by Griess assay. C. OAMSC or RAMSC were collected in Trizol. SOCS1 expression was determined with real-time PCR.

Recent evidence suggests that NO is generated in the inflamed joint in patients with RA. The high amounts of NO production lead to enhanced bone resorption and diminished osteoblast proliferation, decreased proteoglycan synthesis and activated metalloproteases. Our data (Figure 6A) also showed 2–5 fold NO in the joint fluid in patient with RA compared with those in patient with OA. It has been demonstrated that chondrocytes, synovial fibroblasts, and osteoblasts generate NO in vitro in response to inflammatory cytokines. We next examined whether MSCs isolated from joint fluid in patient with RA (RAMSC) contributed to the high NO. Compared with MSCs isolated from bone marrow (BMMSC) of healthy donor RAMSC secreted NO after stimulated with inflammatory cytokines (Figure 6B). We found no detectable levels of NO in BMMSC as reported before [13]. We further tested SOCS1 expression in RAMSC. As shown in Figure 6C, SOCS1 mRNA level in RAMSC was dramatically lower than that in OAMSC.

## Discussion

The immune regulatory properties of MSCs have attracted great attention by both basic and clinical researchers. MSCs have potent immunosuppressive effects on T cells, B cells, DCs, and other inflammatory cells and various molecules have been shown to be responsible [4]. Our studies revealed that SOCS1 regulated the immunomodulatory capacity of MSCs by modulating iNOS expression. With SOCS1 knockdown, MSCs showed enhanced iNOS expression and NO production, which contribute to the strong immunosuppressive activity on T cell proliferation. We also demonstrated the significantly lower SOCS1 expression and higher NO production in MSCs isolating from rheumatoid arthritis patient.

SOCS1 has been found to be expressed in several cells of the immune system, such as macrophages, monocytes and DCs [14]. In the present work we provide evidence, for the first time, that SOCS1 is also significantly up-regulated in both primary MSCs and MSC cell line following exposure to proinflammatory factors. The observed time-course for SOCS1 up-regulation was similar to what was previously described in other immune cells. Although it was initially detected at very low levels in MSCs, upon cell activation the levels of this mRNA increased significantly.

SOCS1 was first characterized as a regulator of IFNγ induced inflammatory responses [24], [25] and recent studies have suggested the control of NFκB signaling [26] and IRF3 driven processes by SOCS1 [27]. Literature has shown that excessive production of TNFα, IL-12, and IFNγ in macrophages, DCs, and fibroblasts from SOCS1-null mice [25], [28], [29]. Our real-time PCR analysis showed the increased TNFα and IFNγ expression in MSCs with SOCS1 knockdown (data not shown).

It has been found that the immunosuppressive ability of MSCs is not innate, but rather is induced by the proinflammatory cytokines, IFNγ in combination with TNFα, IL-1α, or IL-1β [7], [13]. Under sufficiently high levels of proinflammatory cytokines, MSCs increase their expression of iNOS and then NO production. However, in some case, MSCs might encounter insufficient proinflammatory cytokines in vivo. Then the immunosuppressive effect of MSCs might turn off, or even switched to stimulatory effect. Thus, the increase of TNFα and IFNγ expression in MSCs with SOCS1 knockdown might lower the threshold of proinflammatory cytokines and then contribute to the immunosuppressive function of MSCs.

Due to their potential for tissue repair and their immunosuppressive capacities, MSCs have been employed in preventing autoimmune diseases such as RA [30]. However, contrasting results are reported in RA, using the experimental collagen-induced arthritis model [31], [32]. In this study, we demonstrated the significantly lower expression of SOCS1 and higher production of NO in MSCs from the synovial fluid of RA patient. It has been suggested that NO plays a pathogenic role in joint inflammation [33], [34]. Further study is required to test whether the low expression of SOCS1 in MSCs contributes to the high NO production in the synovial fluid. Literatures [35], [36] have shown that different mechanisms account for similar immunosuppression by MSCs in distinct species. In mouse, it is NO produced by iNOS, whereas in human it is tryptophan depletion resulting from IDO upregulation. NO production of MSCs from RA patient might not involve in the immunoregulatory function of the patient MSCs, whether the low SOCS1 expression related to the IDO expression merits further investigation.

Collectively, our study revealed a novel role of SOCS1 in regulating the immune modulatory activities of MSCs and provided some hint for better clinical utilization of MSCs.

## Supporting Information

Figure S1 **Cell proliferation was measured by cell counting analysis and CCK-8 assay.** A, cells at 4000/well were seeded in 24-well plate in cultured medium. Cells were counted every day for 7 days. The mean cell number ± standard error (SD) was calculated for each triplicate. B, cells were suspended at a final concentration of 5000/well in 100 µl medium and cultured in 96-well flatbottomed microplate. CCK-8 (10 µl/well) was added to each well containing 100 µl of culture medium, 4 hours later, OD value were tested and expressed as mean±SD.
(TIF)
