# Supplementary figures and images for: SOCS1 Regulates the Immune Modulatory Properties of Mesenchymal Stem Cells by Inhibiting Nitric Oxide Production
Source: PLoS One. 2014 May 14;9(5):e97256. doi: 10.1371/journal.pone.0097256 (PMC4020773; doi:10.1371/journal.pone.0097256)

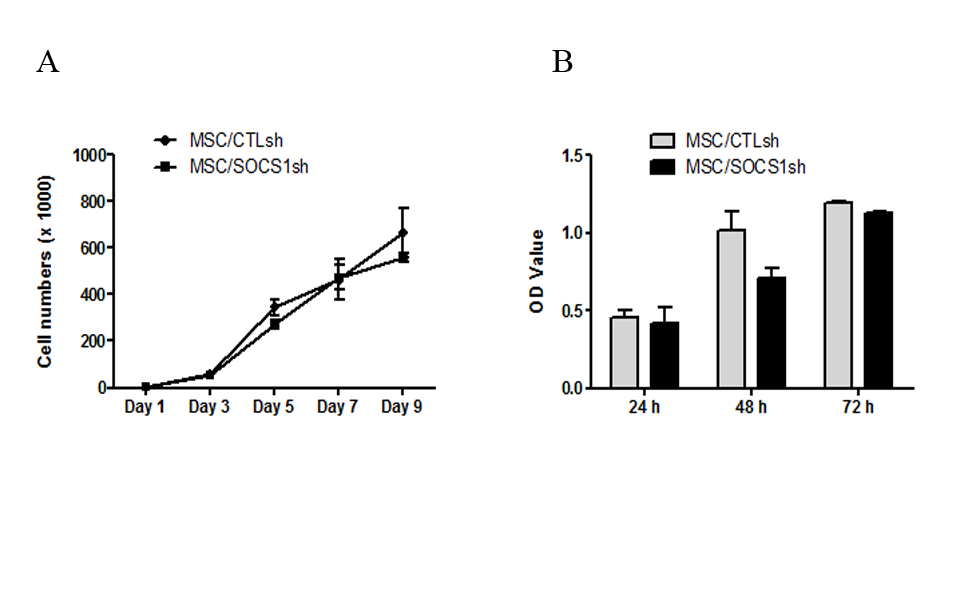

Supplement: Figure S1 — Cell proliferation was measured by cell counting analysis and CCK-8 assay. A, cells at 4000/well were seeded in 24-well plate in cultured medium. Cells were counted every day for 7 days. The mean cell number ± standard error (SD) was calculated for each triplicate. B, cells were suspended at a final concentration of 5000/well in 100 µl medium and cultured in 96-well flatbottomed microplate. CCK-8 (10 µl/well) was added to each well containing 100 µl of culture medium, 4 hours later, OD value were tested and expressed as mean±SD. (TIF) [file pone.0097256.s001.tif]
